# Chronic pain management in fibromyalgia: the INTEGRO (INTEGRated Psychotherapeutic InterventiOn) protocol and its application on two case studies

**DOI:** 10.3389/fmed.2024.1408693

**Published:** 2024-10-24

**Authors:** Ilenia Pasini, Valeria Donisi, Elisa Veneziani, Cinzia Perlini, Marta Nizzero, Irma Lippolis, Enrico Polati, Vittorio Schweiger, Lidia Del Piccolo

**Affiliations:** ^1^Section of Clinical Psychology, Department of Neurosciences, Biomedicine and Movement Sciences, University of Verona, Verona, Italy; ^2^Pain Therapy Centre, Department of Surgery, Dentistry, Maternal and Infant Sciences, Verona University Hospital, Verona, Italy; ^3^Rheumatology Unit, University of Verona, Verona, Italy

**Keywords:** chronic pain management, fibromyalgia, cognitive behavioral therapy, acceptance and commitment therapy, mind–body intervention, self-efficacy, clinical protocol, health-related quality of life

## Abstract

**Objectives:**

To present an innovative integrated manualized psychotherapeutic intervention for fibromyalgia (FM) based on cognitive and behavioral therapy, acceptance and commitment therapy, and somatic experiential techniques (namely the INTEGRated Psychotherapeutic InterventiOn, INTEGRO) and illustrate its application on two case studies.

**Methods:**

INTEGRO is composed of 12 individual sessions. The main objectives of the intervention were psychoeducation of chronic pain mechanisms, understanding the role of cognitive and emotional variables in one’s pain perception, teaching patient-tailored skills to increase pain awareness and its management, and learning how to live with pain experience. A 57-year-old woman (patient A) and a 26-year-old woman (patient B) with FM have been selected to describe their care pathways connected to the INTEGRO protocol. Data related to assessment variables and clinical processes have been reported, focusing on the mechanisms that contribute to the maintenance (i.e., avoidance or overcompensation) of chronic pain in FM, on the role of patients’ naïf theories, and on the implications that all these aspects may have on the burden related to pain management.

**Results:**

Both patients showed a reduction in FM burden and an increase in self-efficacy in pain management: patient A reported an improvement in emotional regulation ability; patient B showed a decrease in pain interference in work activities and on emotional dimension.

**Conclusion:**

Examining each phase of the clinical protocol through the lens of its clinical application, the paper provides insights into the relationship among crucial psychosocial mechanisms, pain perception, management in FM treatment, and how all these aspects have been dealt with during psychotherapeutic treatment.

## 1 Introduction

Fibromyalgia (FM) is one of the prevalent causes of chronic, widespread pain that primarily affects women (3:1, female-to-male ratio) (1). The prevalence of FM varies depending on the diagnostic criteria used to characterize the disorder, although it generally accounts for 2–3% worldwide (2).

Persons with FM report mainly chronic and diffuse musculoskeletal pain, together with a heterogeneous set of complex poly-symptomatology, such as physical and mental fatigue, anxiety and depressive symptoms, sleep disorders, headache, hypersensitivity to external stimuli, and other functional disorders (3–6). However, the clinical presentation might vary significantly within the same person, by context and time, and among persons themselves (7, 8).

The FM pathogenesis is multifactorial and still needs to be clarified. Several factors should be considered as potential disease triggers (9). One recognized hypothesis describes FM as a central sensitization syndrome characterized by the alteration of nociceptive processes (10–12). However, recent findings support the hypothesis that the disease manifests as stress-related dysautonomia with neuropathic pain features (9, 13). Without reliable and easy-to-use biomarkers for daily clinical practice, self-reported instruments are generally utilized to assess this condition (14). The diagnostic process is often long and complex, contributing to patients’ feelings of being invisible, neglected, and “not taken seriously” (15, 16).

The disabling symptoms of FM cause a significant decrease in health-related quality of life (HrQoL), a considerable impact on daily functioning and social interactions, and an increase in emotional distress (17, 18). According to a recent study, the HrQoL categories most affected are “physical pain” and “vitality” (19). Individuals with FM appear to have difficulties in emotion regulation, higher presence of negative affective states, and alterations in enteroception; 60% of persons with FM present a lifetime prevalence of anxiety disorders, while depression is observed in 14–36% of cases (2, 3, 20). Anxiety and depressive symptoms are examples of emotional distress that exacerbates the primary FM symptoms (such as pain, fatigue, and insomnia). This lowers HrQoL and indirectly increases the negative impact of pain on HrQoL (20–22). Moreover, emotional distress, associated with pain-related catastrophic thoughts and fear of pain, contributes to a more intense and aversive pain experience (23, 24). Several studies showed that improved emotional awareness and regulation enhance psychological wellbeing, pain adaptation, positive stress management, and treatment compliance (22, 25–27).

In persons with FM, body perception is characterized by selective and dysfunctional attention to somatic signals, especially those related to painful symptoms, resulting in more significant concerns about their body and avoidance of bodily sensations (28–30).

As regards other psychological variables, self-efficacy and coping strategies have been frequently studied in patients with chronic pain, including FM. Pain-related self-efficacy is associated with better pain adaptation and reduced disability, mediating the effects on the possible development of depressive state symptoms (24, 31, 32). Avoidance, overdoing, and pacing coping strategies result in prevalent approaches to dealing with pain in FM (33). Specifically, pain-related fear leads to avoidance behaviors (34), which, in turn, modify patients’ motor patterns, altering body awareness and reducing physical agility (e.g., loss of balance) (29, 35). Distraction and “overdoing” are two examples of avoidance behaviors, whereas the ‘pacing’ coping strategy, defined as an “activity–rest” cycle or “slow-but-steady” movement (36), appears to be adaptive for chronic pain management (33). Distraction without reframing what happened negatively impacts the severity of the perceived pain (28, 37). Excessive persistence or “overdoing” means that the activity is prolonged or performed at a higher intensity than the patient can tolerate (i.e., when perseverance in the activity permits a task to be completed without a flare-up of discomfort, this appears to be a functional approach; on the contrary, it represents a maladaptive overdoing) (33, 38).

Taking into account the characteristics of FM, treatment should be tailored to the patient and based on a biopsychosocial approach by integrating different components, such as pharmacological and psychosocial treatment, as well as physical activity (39–41).

A growing body of evidence supports the effectiveness of psychological therapy in managing the wide range of cognitive, emotional, and behavioral symptoms associated with FM, with a relevant role for clinical psychologists in the multidisciplinary team for FM treatment. Given the critical role of psychological variables in FM (42), psychotherapy may be beneficial for treating chronic pain (40, 43, 44), with some approaches being especially effective in improving emotion regulation competencies and functional pain-related coping strategies. Specifically, cognitive behavioral therapy (CBT) has been shown to reduce painful symptoms and negative mood deflection and to improve HrQoL and self-efficacy (45, 46) in pain management of FM patients. Acceptance and commitment therapy (ACT) has been shown to enhance the acceptance of pain and reduce pain catastrophizing (47). Body-oriented psychotherapy interventions (i.e., mind–body interventions, embodied cognition approach, and body awareness therapy) also seem to have a positive effect on the management of somatic symptoms related to chronic pain (48) and fibromyalgia syndrome (49, 50). Recently, in the management of FM, practices focused on embodied cognition, based on movement or the perception of it and aimed at reestablishing sensorimotor integration has been considered crucial for fostering reconnection with bodily sensations, promoting a confident and non-judgmental view of one’s body (29).

Given the documented relevance and benefits of CBT, ACT, and the recent interest in embodied cognition approaches for pain management, the INTEGRated psychotherapeutic intervention (namely INTEGRO) protocol has been created to help persons with fibromyalgia manage chronic pain (51). The INTEGRO protocol has the peculiarity of integrating, in a manualized treatment, evidence-based practices that help FM patients deal with pain to achieve the following targets:

- To reduce the impact of fibromyalgia symptoms on daily activities by improving HrQoL,
- To lower pain intensity perception,
- To increase perceived self-efficacy in pain management,
- To improve emotional regulation skills.

This study aimed to: (i) describe in detail all steps and topics of the INTEGRO intervention; (ii) show how the implementation of multimodal pain management in clinical practice can be organized by describing the INTEGRO application to two different cases, prototypical of FM patients; (iii) report how the intervention impacts on HrQoL, pain perception, pain-related coping strategies and perceived self-efficacy, psychosocial mechanisms related to pain, emotional regulation skills and body awareness in each of the two patients.

## 2 Materials and methods

### 2.1 Procedure

The INTEGRO study ‘INTEGRated Psychotherapeutic InterventiOn’ is an exploratory longitudinal prospective study (see Pasini et al. (51) for a complete description of the study protocol) and is based on the collaboration between the Clinical Psychological Unit, the Pain Unit, and the Rheumatology Unit of Verona Hospital (Azienda Ospedaliera Universitaria Integrata—AOUI). The study has been approved by the Ethical Committee of Verona Hospital (Prot n. 54513, 12/09/2022). The medical staff of the Pain Unit and the Rheumatology Unit recruit patients who meet the inclusion criteria (i.e., FM diagnosis according to established ACR criteria—American College of Rheumatology (52), and idiopathic chronic pain; 18–65 years old; Italian-speaking; able to provide informed consent).

After being selected, patients sign the informed consent form before participating.

**Figure 1:**
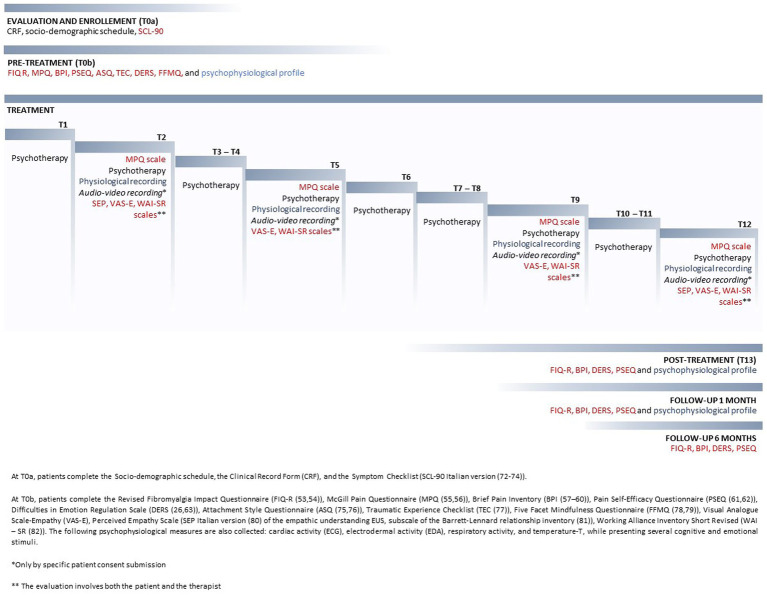
Timeline and procedure of the INTEGRO protocol.

The timeline and procedure of the INTEGRO protocol are reported in detail in Figure 1.

### 2.2 INTEGRO assessment

Each patient was assessed by using the following instruments:

- Revised Fibromyalgia Impact Questionnaire (FIQ-R) Italian version (53, 54) to evaluate functioning, symptoms, and impact on daily activities (HrQoL) of FM;
- McGill Pain Questionnaire (MPQ) Italian version (55, 56) and the Brief Pain Inventory (BPI) Italian version (57–60) to measure pain intensity perception and different components of pain;
- Pain Self-Efficacy Questionnaire (PSEQ) Italian version (61, 62) to evaluate self-efficacy in chronic pain management;
- Difficulties in Emotion Regulation Scale (DERS) Italian version (26, 63) to assess emotion dysregulation and the acquired skills to reduce it.

The evaluation of pain using the MPQ has been performed at T0, T2, T5, T9, and T12.

The pre- and post-assessment of the other psychosocial variables was conducted before the intervention (T0) and 1 week before the end of treatment (T13).

For more details on all questionnaires adopted in the INTEGRO study, see Pasini et al. (51).

### 2.3 Description of the INTEGRO intervention

INTEGRO is implemented in the Clinical Psychology Unit of the Verona University Hospital, Italy, and is led by two clinical psychologists skilled in CBT and ACT and trained in the application of relaxation and mindfulness-based approaches with expertise in chronic pain management.

The intervention is structured in three phases and comprises 12 sessions of 1 h each, performed every 7 or 15 days, according to the patient’s needs. Clinical psychologists manage the intervention according to a manualized protocol containing specific aims, topics, and exercises for each session.

The principal steps of the intervention are described in Table 1.

**Table 1:** INTEGRO intervention’s main characteristics.

**Phase 1—Engagement, Motivation, Psychoeducation**
Sessions 1–2—**Description of pain history, theories, and experiences related to disease and introduction to pain mechanisms**. **Promote pain awareness** using the **McGill Pain Questionnaire (MPQ)** and pain monitoring log (i.e., antecedent to the onset of the painful symptom, possible triggering factors, related thoughts, emotions and physical sensations, pain reaction, and outcomes). **Psychoeducation on pain** (e.g., evolutive functions of pain, sensory, cognitive, affective, and behavioral components of pain, mechanisms of functioning) that aims to help the patient recognize pain components through a specific pain monitoring log (created *ad hoc* for INTEGRO).
Sessions 3—**Psychoeducation on chronic pain** (e.g., the difference between acute and chronic pain, central sensitization and alterations of pain inhibitory mechanisms, the role of perception in the experience of pain). **To promote awareness of pain-related cognitive mechanisms** by using a pain monitoring log (created *ad hoc* for INTEGRO).
Session 4—**Pain maintenance mechanisms** (i.e., presence of pain management strategies based on the control paradigm, pain maintenance cycles related to discouragement/depression, anger, anxiety, and fear, both using illustrative graphic representations and through the construction of one’s schemes)
Session 5—**Pain communication** (i.e., the patient learns how to communicate one’s needs and states of distress, interpersonal cycles of rejection, and distancing or over-caring) Self-awareness of interpersonal cycles can be achieved using graphical representations (forms created *ad hoc* for INTEGRO). Promote awareness of current pain using the **MPQ** Scale.
Session 6—**Coping strategies** (i.e., the patient becomes aware of personal coping strategies and protective behaviors by using graphic representations showing stress-pain and vicious maintenance cycles) (e.g., rumination, hyperarousal, and avoidance) (forms created *ad hoc* for INTEGRO). **Learning the distinction between “clean” pain** (“nociceptive pain”) **and “dirty” pain** (the subjective sensation related to the pain that inhibits our life) through the patient’s reports and pain monitoring log [forms created *ad hoc* for INTEGRO and partially modified from Joann and Lundgren (69)].
**Phase 2**—**Cognitive Restructuring, Avoidance Reduction, Promotion of Alternative Behavioral Strategies, Experiential Awareness Techniques, and Defusion**
Session 7—**Body awareness** (i.e., to promote the distinction between “clean” and “dirty” pain using a specific log in which the patient describes the situational antecedent, clean pain vs. dirty pain intensity; to reduce avoidance and to promote alternative behavioral strategies; to introduce body awareness techniques to recognize and distinguish somatic signals with the aim of appropriately regulate them). **To help the patient understand her relationship with her body** regarding emotions and sensations, she should observe what can be perceived through the body (without focusing on painful sensations) and use grounding resources [adapted from Ogden and Fisher (70)].
Session 8—**Awareness of body sensations to improve relaxation skills** (i.e., psychoeducation on the physiological component of emotions to improve awareness of bodily sensations and their variation in stressful situations) **Embodied techniques** to improve relaxation skills focused on body temperature.
Session 9—**Value orientation** (i.e., to help the patient identify her values and actions in line with her values). To promote awareness of current pain using the **MPQ Scale**, evaluating how the ability to recognize bodily sensations is evolving.
Session 10—**Cognitive defusing to promote pain acceptance** **Use of embodied techniques focused on the breath** to identify somatic centering resources [adapted from Ogden and Fisher (70)]. **Introduction of embodied techniques to explore the qualities of pain** and not be judgmental toward pain [forms created *ad hoc* for INTEGRO and partially modified from Marchi and Blasutti (71)].
Session 11—**Pain acceptance** (i.e., cognitive defusing using a pain log) [forms created *ad hoc* for INTEGRO and partially modified from Joann and Lundgren (69)]. **Embodied techniques to accept pain**, welcoming pain in its characteristics, observing it without judgment, and guiding the breath through it.
**Phase 3**—**Conclusion**
Session 12—**Definition of own toolkit for pain management** (i.e., sharing of the objectives achieved during the treatment and identification of the tools acquired; consideration of the difference between acceptance and resignation, also through an imaginative exercise) Value the awareness of current pain using the **MPQ Scale** and how it is evolving, underlining the changeability of pain and the skills acquired by the patient to distinguish pain components.

### 2.4 Description of the two patients selected to explain how INTEGRO protocol works

Two patients were selected from the study to show how different strategies for dealing with FM symptoms can be managed within the INTEGRO protocol.

Case 1—Patient A:

- Patient A is a 57-year-old woman who lives alone and is unmarried. She works as a professional nurse. FM was diagnosed when she was 55 years old, although the pain began 2 years earlier.
- *Characteristics of pain and fibromyalgia*: Reported symptoms included poly-district pain, the continuous and diffuse hitch that primarily affected the lower and upper limbs, pelvic area, and craniofacial area; severe asthenia; muscle fatigue; cognitive deficit (reduction in concentration, memory, and attention); non-restorative sleep; paresthesia; significant qualitative changes in vision; poor tolerance to various foods; constant profuse sweats accompanied by nausea; and a feeling of deep anguish.
- *Medical–surgical history*: Clinical history is characterized by significant psychological suffering as a result of traumatic events (in childhood and early adulthood), mechanistic arthropathy (polyarthritis) supported by the effects of ligamentous hyperlaxity-type Ehlers–Danlos syndrome, spondylolysis, spondylolisthesis (L5/S1) due to recurrent lumbago; bilateral gonarthrotic pain, musculoskeletal headache, and migraine with aura (adolescent onset), three traumatic brain injuries; removal of myxoid liposarcoma in the right lower limb and local radiotherapy, with the removal of the soleus muscle and sclerosis of the surrounding soft tissues. The subsequent alteration of the skeletal alignment has accentuated the preexisting widespread pain in the compensatory postural phase and made it necessary to use a walking aid.
- *Pharmacological and non-pharmacological treatments and their efficacy*: The patient underwent cycles of hydrokinetic therapy and free body gymnastics for deep muscle building, with moderate results. She is treated with duloxetine (60 mg + 30 mg), quetiapine (25 mg), pregabalin (3× 25 mg), and ibuprofen at need, with 50–70% perceived pain relief.
- *Functional, emotional, and cognitive impact on the patient*: Patient A is currently on sick leave. She plans to apply for early retirement due to a significant impairment in her ability to perform daily activities (e.g., she is unable to drive autonomously and requires specific assistive devices for mobility). Friends give her social support, whereas her brother rarely does.

Case 2—Patient B:

- Patient B is a 26-year-old woman who works as a clerk, is single, and lives with her parents. The diagnosis of FM was confirmed in 2021, but the onset of pain traces back to childhood and worsened during the previous year.
- *Characteristics of pain and fibromyalgia:* Reported symptoms include widespread pain, specifically in the cephalic and cervical area, upper limbs, and rarely in lower limbs, chronic pelvic pain, lumbago, fatigue, cognitive impairment, non-restorative sleep, the feeling of swelling in the hands, auditory sensory losses, and poor tolerance to various foods.
- *Medical–surgical history*: clinical history is characterized by numerous admissions to the Emergency Service without apparent evidence, a significant episode of psychological suffering due to a traumatic event (in early adulthood); irritable bowel syndrome, medically unexplained ligamentous laxity, cervical C3-C4 disk protrusions in the absence of radiculopathy, adenomyosis in extra-progestin therapy, bilateral labyrinthitis, and infectious mononucleosis in 2018. She also underwent surgery for adenoidectomy and bilateral inguinal hernioplasty at a young age. In 2021, a few months after the diagnosis of FM, she reported a minor road injury that was followed by a minor cervical distractive injury.
- *Pharmacological and non-pharmacological treatments and their efficacy*: The patient underwent several physical therapies, such as a cycle of magnetotherapy, without any efficacy. She is treated with therapeutic cannabis—CBD 8–10% (10 drops daily use), Paracetamol (1,000 mg daily use), Diazepam, and NSAIDs at need, with 60% perceived pain relief.
- *Functional, emotional, and cognitive impact on the patient*: Parents give her solid social support, while she feels discouraged and fears judgment from friends and colleagues.

## 3 Results

### 3.1 Changes observed in the two selected patients during the INTEGRO therapeutic intervention

This section describes the clinical progression of each selected patient by reporting the main qualitative changes according to the topics and the steps that define the INTEGRO intervention: pain description and perception, pain mechanisms and coping strategies to manage pain, pain and interpersonal relationships, exploration of “clean and dirty pain,” body awareness, pain acceptance, cognitive defusing, and value orientation. Differences and commonalities among patient A and patient B for each thematic area of the intervention have been detailed.

#### 3.1.1 Pain description and perception

Patient A experienced intense pain during the morning, with greater rigidity and gradual worsening throughout the day. Going to work or engaging in any activity requiring continuous hand use and/or physical activity (such as shopping, gardening, or physical exercise) worsened the pain.

In patient B, the pain reached the maximum intensity toward evening; at this time of the day, she felt wholly rigid and contracted. When the same posture was held for more than 15 to 20 min, the pain worsened, and it was frequently necessary to stand up or remain in a standing position. This phenomenon also occurred throughout the INTEGRO intervention sessions. Even routine tasks like smiling, blow drying their hair, and tanning determined an increase in the intensity of pain.

#### 3.1.2 Pain mechanisms and coping strategies to manage pain

When discussing approaches to coping and pain mechanisms in sessions 3–6, both patients reported pain management strategies such as overinvestment or avoidance. However, they used those coping strategies with a different frequency.

In patient A, the main pain-coping strategies were ignoring the pain and persisting with tasks beyond the perceived limit, rest, distraction, containing and demoralizing, and medication use (even with preventive purposes). The most used strategy was overinvestment in managing pain, rigidly continuing with the activity she was doing even at the cost of worsening the pain intensity. In these situations, she was aware that she had reached physical limits (i.e., physical fatigue and pain in her hands) but did not stop, addressing herself with anger and criticism to continue the activity (i.e., by saying to herself: “*You have no excuse”; “You’re just a lazy brat*”) and subsequently reporting physical exhaustion, depressed mood, and inability to move limbs due to perceived pain. Continuous ruminating on how frequently she had accomplished her objectives resulted in greater arousal, muscle stiffness, tension, a sense of the head on fire, and torn muscles. This process made it difficult for the patient to manage her resources, causing her to avoid activities relevant to her health. A significant amount of time had to be spent within the clinical protocol examining and comprehending the pain-related vicious cycles, emphasizing the cost associated with the patient’s overdoing mechanisms. Only in the last sessions did patient A gain the ability to recognize her overdoing mechanism and to decrease the tendency to apply it as an automatic mechanism.

The prevalent pain management strategies of patient B included ignoring the pain, avoiding behaviors, using medication, increasing physical activity, and controlling nutrition. She was inclined to stay away from social events because of worry that she would become unwell in an environment where no one was familiar with her disease. Indeed, in these situations, the fear of being judged as different and of little value prevailed. Avoiding social situations was also prompted by dysfunctional thoughts about the possible consequences: “*If others see that I am sick, they will exclude me*.” This increased the sense of frustration and the idea of being in the “*prison of pain*.” Such thoughts were related to feelings of panic, anxiety, and fear and, subsequently, to a significant increase in excitement, muscle tension, and physical stiffness, which, in turn, amplified the perceived pain. Significant avoidance of body signals, including pain, was also present to pander to the primary need for social approval. This pattern was also evident in ignoring body awareness exercises performed during sessions. In the subsequent sessions, patient B gained the ability to recognize painful avoidance behaviors and catastrophic thoughts through pain monitoring exercises. By recognizing her pain management strategies, she was able to think through the effects of these processes and begin to employ alternative coping strategies (i.e., exposing herself to fearful situations and not preemptively giving up on pleasant experiences).

#### 3.1.3 Pain and interpersonal relationships

Patient A focused on caring for others rather than herself and did not perceive it possible to ask others for help. She could share thoughts about FM only in the friend network, but she did not ask for support due to the fear of being judged negatively. This mechanism progressively increased social withdrawal, self-criticism, poor self-efficacy, sadness, and anger toward herself. The only people the patient felt she could share her experience with were healthcare professionals.

Patient B tended to rely heavily on family care, showing an addictive attitude toward them, triggering a cycle of overcaring, thus strengthening the idea of not being able to manage the pain independently. Toward friends and colleagues, she presented feelings of distrust mainly related to the fear of not being understood, rescued, or being judged weak. This attitude reinforced the need to control the pain symptoms, resulting in an increased likelihood of experiencing anxiety, as well as social isolation and reduced positive experiences.

Both patients reported a history of invalidation of their pain by significant ones. Therefore, in all subsequent intervention sessions, it was necessary to pay attention to interpersonal issues (tendency toward selflessness in patient A and social, emotional, and experiential avoidance in patient B). Both patients showed greater awareness of their interpersonal patterns and an initial drive for change during the therapeutic sessions.

#### 3.1.4 Exploration of pain: “*clean and dirty pain*”

Patient A quickly learned to identify the “*dirty pain*” component (e.g., in the constant judgmental and self-punishing demands she made on herself) and its consequences in terms of increased perceived pain and negative impact on daily functioning. Interestingly, in later sessions of the clinical protocol, the patient reported moments in which she did not recognize an initial ‘*clean*’ component of pain. Still, she was able to recognize the emotional component defined by a sense of helplessness, overwhelm, and high anxiety. This key could also be related to the naïve theory of her illness: the belief that the emotional component played an essential role in the symptomatologic onset and maintenance of pain.

Patient B’s naïve theory of her illness was mainly based on organic causes without an apparent symptomatologic onset. The patient tried not to pay attention to her pain, being afraid of identifying its presence. This was also evident in the inconsistent completion of the “*clean and dirty pain*” diary between the INTEGRO sessions. Despite the difficulty of distinguishing between the emotional and physical components of pain, especially when it was very intense, the patient was able to identify dirty pain in fear reactions and in the tendency to run away. Therefore, during the treatment, special attention was given to the distinction between the two components of “*clean and dirty*” pain by helping the patient recognize those components and their related emotional function.

#### 3.1.5 Body awareness

At the beginning of phase 2, both patients expressed initial skepticism about the feasibility of performing activities that entailed “*‘being’ with one’s body without seeking to avoid any sensations*” as suggested by the ACT approach. Indeed, for both patients listening to physical sensations was directly associated with pain perception. This belief that observing physical stimuli could contribute to pain-increased perception was associated with anxiety feelings (which caused panic attacks in patient B). Moreover, looking at other factors associated with pain, in both cases, the painful perception was exacerbated by environmental stimuli (e.g., poor light conditions, cold, and humidity) or emotional factors (e.g., anxiety or fear; depressed mood).

Despite these difficulties, patient A tried to listen to body sensations, at least in situations not perceived as risky. For example, after a few sessions, she could focus on and describe the pain in her leg: *‘I have the feeling that I can see myself inside the tissues, the ligaments as if I can observe fiber by fiber...I can move freely in this space...I sit in a kind of neutral basin but close enough to the area of pain: it’s a ‘big dark, dense, molasses-like mass, dangling between tissues...it sticks here and there...*’

Patient B, especially in the first sessions, struggled to stay in touch with her bodily sensations (i.e., stating that the more she tried to relax, the more her body stiffened and felt a sense of nausea). She tended to control her internal states because of the fear that something irreparable might happen. Although she benefited from the proposed exercises, she tended to become distracted in the first few sessions and did not persist in practicing them at home. During the first exercises to manage pain, she reported: *‘I cannot be around it; this damn nausea overrides everything... it disgusts me.’* Only as the exercises progressed did the patient consider that she could observe what was happening in her body without feeling the need to control it, fostering acceptance of the pain: *“By focusing on the breath, I can imagine the pain flowing through the body.”*

#### 3.1.6 Pain acceptance, cognitive defusion, and value orientation

By the end of phase 2, patient A improved awareness of how some actions ideally designed to protect her from pain instead moved her away from a life based on desired values. By using “*defusing*” strategies on thoughts related to dirty pain, she learned to slow down when she perceived a painful sensation, to be in contact with the pain and have a clear, non-fearful representation of pain, and to perceive herself as able to continue to live with the pain and carry out, according to her limitations, acting in line with her values.

Patient B realized how obsessively her life focused on preventing pain (perceived as a prison that distanced her from the freedom to choose her own life). The intervention helped her to identify the costs of this struggle and to distance herself from thoughts and emotions related to “dirty pain.” By embracing a certain amount of risk and rediscovering some of the previously avoided life events, it was possible to tolerate the unpredictable nature of pain and increase one’s self-efficacy while dealing with it.

In the last sessions, both patients showed an improvement in cognitive “*defusing*” ability by accepting pain as a disturbing but not limiting presence. This awareness allowed them to actively identify which strategies could be more functional for specific pain experiences. Patient A reported an improvement in self-care and pacing strategies, a decrease in dysfunctional overdoing, and preventive use of drugs by becoming aware that she could “shape her pain through experiential techniques.” Patient B showed a reduction of avoidance and increased social exposure, assertive communication of their own needs, and the use of relaxation techniques to reduce the perception of pain.

### 3.2 Evaluations along the INTEGRO intervention

This section describes quantitative and qualitative variations in INTEGRO measures.

#### 3.2.1 Pain intensity and quality

**Figure 2:**
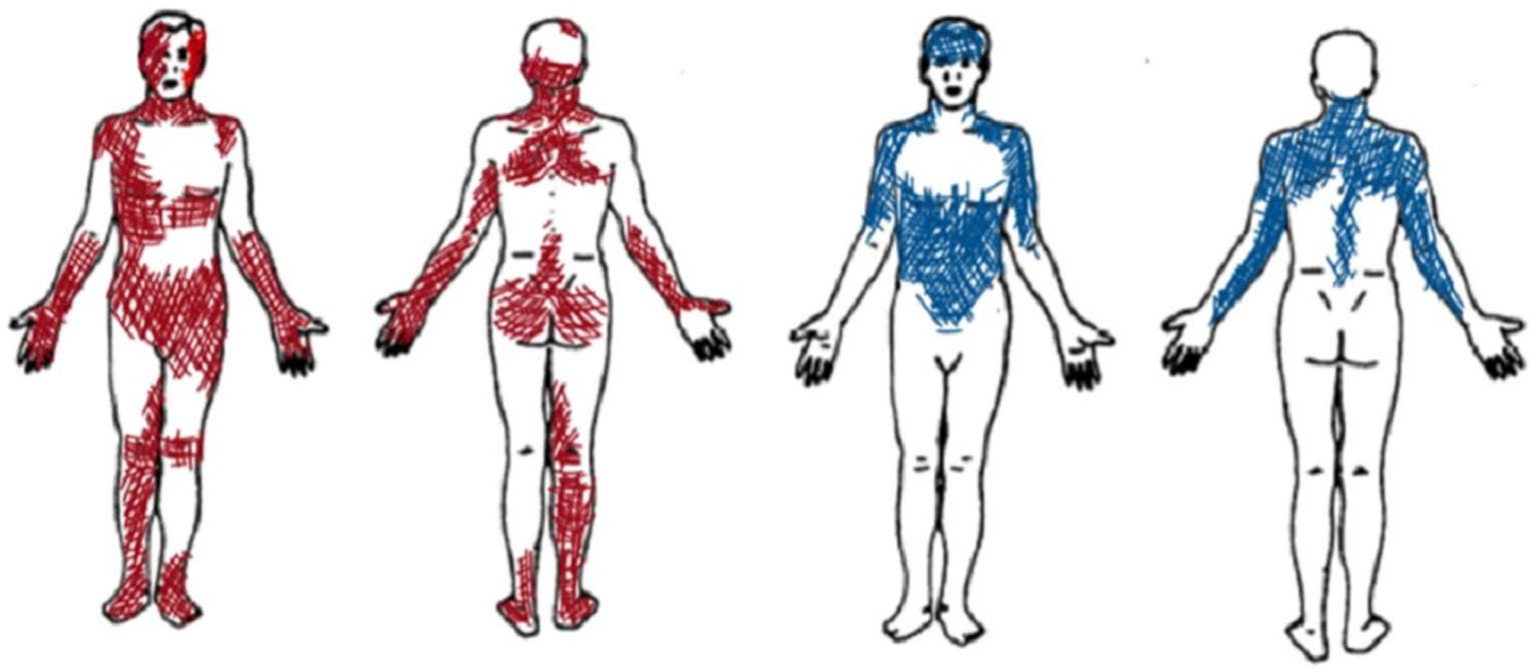
Graphic representation of pain localization as reported by Patient A (in red) and Patient B (in blue).

In the first sessions, patient A reported large and overlapping pain locations (sessions 1 and 2) (see Figure 2). She used a wide range of pain descriptors (i.e., Number of Words Chosen—NWC) in the MPQ Scale (such as flickering, jumping, pricking, tender, exhausting, sickening, fearful, and wretched). She also described the pain in the hands as ‘flaming flows’, while pain in the feet as “moving insects.” Contact with surfaces caused pain, and sitting on the bed or in chairs was difficult. The difficulty of choosing certain words or body parts appeared to be related to challenges relevant to pain moment-by-moment awareness, which decreased during the therapeutic sessions.

Since the beginning, patient B selected specific pain descriptors and narrowly defined body areas (see Figure 2). During the therapeutic sessions, she selected similar descriptors of the MPQ Scale (mainly using sensory descriptors such as aching, tender, pulling, tiring, troublesome, pulsing or beating, and pressing or crushing). She indicated similar pain areas and intensity, thus suggesting a constant type of pain perception over time.

**Figure 3:**
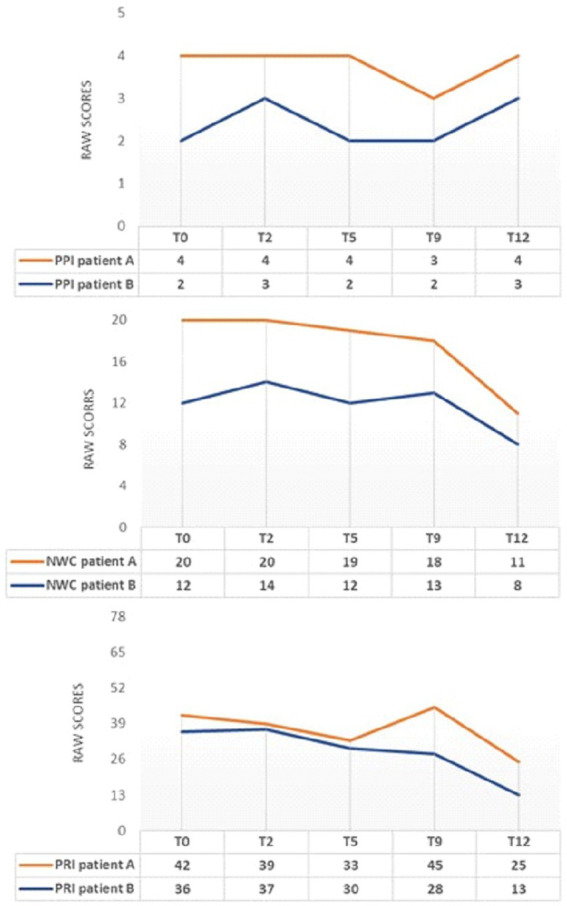
MPQ changes during INTEGRO evaluation sessions (T0-pre-treatment evaluation; T2, T5, T9-Intermediate treatment evaluations; T12-final treatment evaluation). PPI=Present Pain Intensity (Score range 0-5)is a numeric-verbal combination that indicates overall pain intensity rated on a 6- point Likert scale ranging from ‘none’(0) to ‘atrocious’(5); NWC=Number of Words Chosen (Score range 0–20), this index represents the number of words used to describe pain; PRI-Pain Rating Index (Score range 0–78),the score is based on position or order of rank in the set of words and describe a qualitative pain perception (55, 56).

Figure 3 describes pain using MPQ and how it varied during sessions.

Both patients A and B show a general decrease in the indexes of PRI and NWC.

Note that patient A shows an increase in the PRI index at T9. This increase in pain rating does not correspond to a worsening perceived pain (PPI = 3) or the evaluative dimension (score = 0.8). Still, it may be a consequence of the scoring related to the sensory dimension, which may occasionally change due to the use of worse words to describe pain by the patient.

Focusing on the description of pain during the encounters led both patients to consider pain as changing in intensity and not necessarily being the same over hours and days.

**Figure 4:**
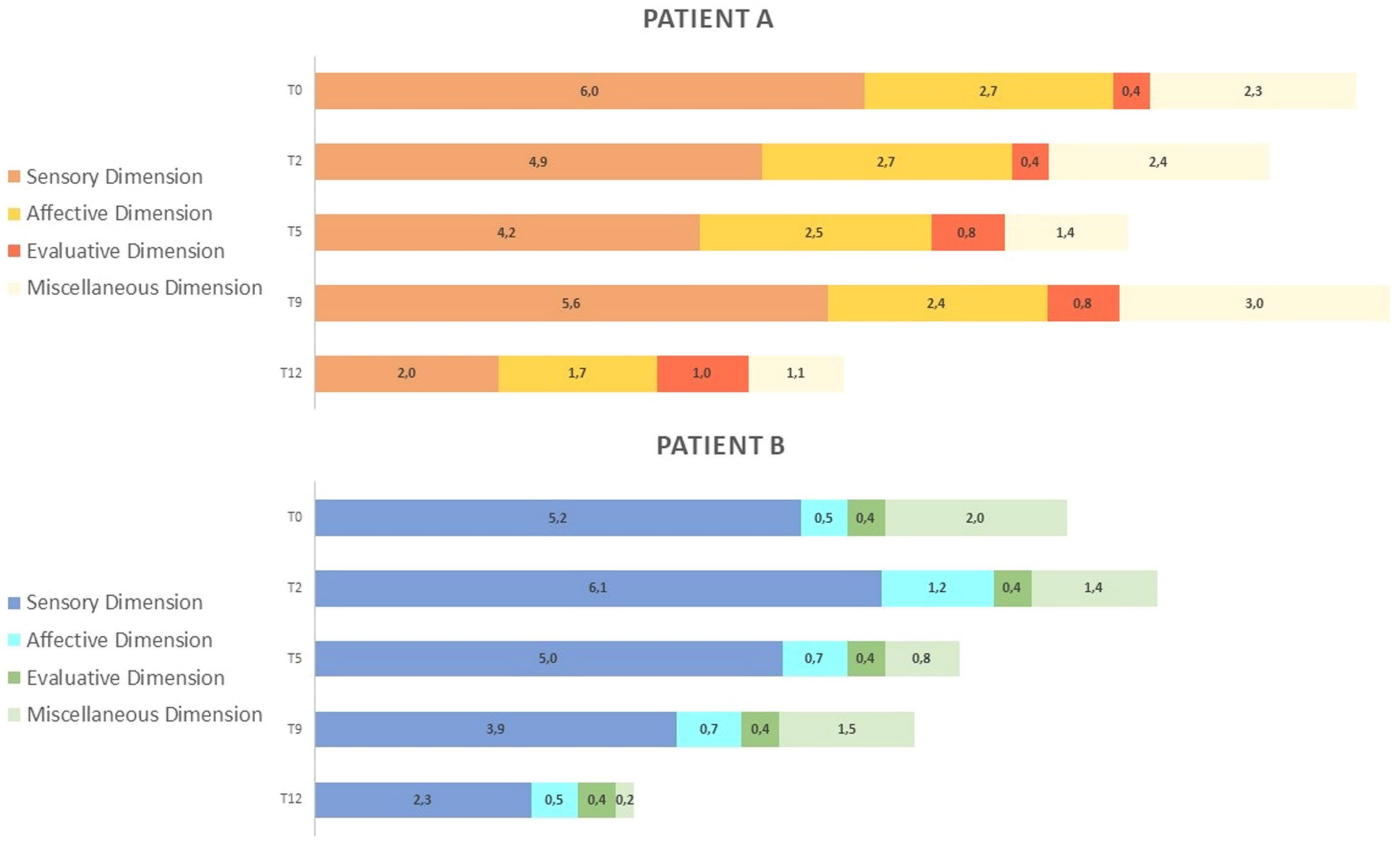
(A) MPQ scores in the three dimensions: Pain Sensory, Pain Affective, Pain Evaluative across INTEGRO measurement sessions (T0-pre-treatment evaluation; T2, T5, T9-intermediate treatment evaluations; T12-final treatment evaluations). The Sensory dimension (range 0-10) reports temporal, spatial, pressure, thermal, and other sensory properties of perceived pain. The Affective dimension (range 0-5) reflects tension, fear, emotional aspects of pain and automatic components. The Evaluative dimension (range 0–1) informs on the subjective overall intensity of experienced pain. (B) MPQ distribution over time (T0-pre treatment evaluation;T2,T5,T9- intermediate treatment evaluations; T12-final treatment evaluation) in dimensions: Pain sensory, Pain affective, Pain evaluative, Miscellaneous (range 0-4)which includes words that are often chosen but do not refer to any specific dimension.

Figure 4 reports McGill Pain Questionnaire (MPQ) scores in the three dimensions: Pain Sensory, Pain Affective, and Pain Evaluative, across INTEGRO measurement sessions (T0—pre-treatment evaluation; T2, T5, and T9—intermediate treatment evaluations; T12—final treatment evaluation).

Patient A shows increased sensorial component (score 5.6) and miscellaneous (score 3.0) at T9. Both patients show a reduction in scores on the sensory and affective subscales (patient A score in the sensory dimension is 2.0 and that in the affective dimension is 1.7; patient B scores are 2.3 and 0.5, respectively).

Patient A shows an increase in evaluative dimension (from a T2 score of 0.4 to a T12 score of 1), whereas patient B shows no variation over time.

Section b of Figure 4 highlights how the proportion of pain dimensions changes along the intervention. Both patients show a reduction and a redistribution of sensory and affective components at the end of the intervention.

#### 3.2.2 Pre (T0)- and post (T13)-intervention assessment: fibromyalgia interference, pain intensity, perception of self-efficacy, and emotional regulation

Table 2 shows the results of clinical assessment at T0 and T13.

**Table 2:** Results of clinical assessment Pre-treatment (T0) and post-treatment (T13).

	**Patient A**		**Patient B**	
	T0	T13	T0	T13
Revised Fibromyalgia Impact Questionnaire—FIQ				
Total score (score range 0–100)	93.8	84	77.3	53
Physical function (score range 0–30)	28.3	28	21.3	16
Overall impact (score range 0–20)	20	18	11	0
Symptoms (score range 0–50)	45.5	38	45	37
Brief Pain Inventory—BPI				
Pain intensity (score range 0–10)	8.6	7.8	6.4	7
Emotional interference (score range 0–10)	9.3	8.7	7	4.7
Work interference (score 0–10)	9.7	10	6	2
Pain Self-Efficacy Questionnaire—PSEQ				
Pain self-efficacy (score range 0–60)	9	11	17	45
Difficulties in Emotion Regulation Scale—DERS				
Total score (score range 33–165)	131	98	89	89
Non-acceptance (score range 6–30)	28	13	10	13
Goals (score range 5–25)	21	21	19	19
Impulse (score range 6–30)	27	17	22	18
Awareness (score range 3–15)	7	9	3	4
Strategies (score range 8–40)	28	22	25	24
Clarity (score range 5–25)	20	16	10	11

For each scale, minimum and maximum score variations are reported. For FIQ, lower scores indicate a lower impact of FM; for BPI and PSEQ, higher scores indicate higher interference of pain and perceived self-efficacy in pain management, respectively; for DERS, higher scores indicate more significant difficulties in emotional regulation.

As for FM interference, both patients showed an improvement in the total score of FIQ (patient A ranged from 93.8 to 84; patient B ranged from 77.3 to 53) as well as for the Physical function subscale (patient A ranged from 28.3 to 28; patient B ranged from 21.3 to 16), Overall impact (patient A ranged from 20 to 18; patient B ranged from 11 to 0), and Symptoms (patient A ranged from 45.5 to 38; patient B ranged from 45 to 37). In patient B, the change at T13 in total score was high enough to result in a change in severity status from “severe disease” (defined as a score range of 64–82) to “moderate disease” (defined as a score range of 41–63) according to the scores reported by Salaffi et al. (14).

As for the intensity and impact of pain (BPI questionnaire) during the previous 24 h, a slight reduction in pain intensity in patient A emerged (from a score of 8.6 to 7.8), while the dimensions of emotional interference and work interference were stable. In patient B, there is a reduction in pain interference on both the emotional dimension (from a score of 7, indicating a severe degree of interference, to 4.7, indicating a moderate degree of interference) and work–life activities (from a score of 6, indicating a low–moderate degree of interference to 2, indicating a mild low degree of interference) after the intervention.

As for self-efficacy (PSEQ questionnaire), in patient B a relevant increased sense of self-efficacy in pain management after the intervention was evident (from a score of 17 to 45), while patient A showed only a slight increase (from a score of 9 to 11).

The Emotional Regulation (DERS questionnaire) Scale improved in patient A, as evidenced by a decline in total scores (from a score of 131 to 98) and the subscale capacity to accept emotional responses (Non-acceptance T0 score of 28; T13 score of 13), impulse control (Impulse T0 score of 27; T13 score of 17), access to emotion regulation strategies (Strategies T0 score of 28; T13 score of 22), and emotional clarity (Clarity T0 score of 20; T13 score of 16).

Patient B reported a slight decrease in the Impulse subscale scores (Impulse T0 score of 22; T13 score of 18) and no significant changes in the other scores.

## 4 Discussion

Most protocols in the literature for the management of fibromyalgia syndrome tend to focus on standard cognitive behavioral therapies or individually implemented approaches in group sessions (64) The INTEGRO protocol integrates different strategies and techniques that draw on various methods. It also modulates the individual sessions’ content based on the patients’ experiences, making it possible to create a flexible intervention focused on the most problematic areas. It is also important to highlight how INTEGRO protocol integrates with treatments of a more medical nature and can easily be combined with rehabilitative interventions.

Examining each phase of the clinical INTEGRO protocol through the lens of its clinical application permitted us to comprehend (i) the relationship between various psychosocial factors and FM pain management and (ii) the process of change on which clinicians had to pay attention to consent the adaptation of INTEGRO intervention to clinical issues related to each patient’s peculiarities.

Major FM patient characteristics were evident in both cases, representing an example of the wide range of symptoms and psychosocial mechanisms influencing pain and HrQoL (7, 8) in FM.

Patient A and patient B showed relevant differences in terms of:

1. Pain perception: more diffuse, with a peak during morning times and a higher affective component in patient A, and more selective, with a peak during evening times and with a prevalent avoiding attitude in patient B;
2. Psychosocial context: both were single, but patient A lived alone and could rely on a good network of friends, even if she did not share her pain-related worries; patient B lived with her parents and was very demanding of them;
3. Psychological functioning: patient A tended to blame herself when she was unable to gain her aims and tended to deny her needs; patient B tended to avoid feelings, paying attention mainly to bodily sensations and showing difficulties in mentalization processes;
4. Naïve explanation of illness: patient A tended to find a strong connection between emotional condition and physical response; patient B sought an explanation only through organic miens;
5. Coping strategies: patient A persisted with tasks beyond the perceived limit, iper-invested in managing pain, and tried to prevent it by using several painkillers; patient B tried to use mainly control strategies, giving up when they were no longer effective with avoidance attitudes.

Despite these differences, after the INTEGRO intervention, both patients showed a reduction in the burden of FM as measured by the Revised Fibromyalgia Impact Questionnaire—FIQ, even if only patient B reported a significant change from the “severe disease” to the “moderate disease” category.

The improvement in health-related quality of life, despite the severity of physical disease, was related to the specific characteristics of each patient as has been evidenced by the combined use of many tools for assessing pain. This approach permitted a better investigation of the changes that have taken place during the intervention as well as a deeper analysis of the mechanisms on which to act to enhance positive outcomes.

Patient A started treatment with the idea that the onset and worsening of her symptoms were strongly interconnected with emotional stress and traumatic experiences, patient B tended to mainly trace the cause to previous medical conditions. Therefore, patient A showed much more sensitivity to all practices related to self-awareness, recognizing the progress obtained and feeling legitimated in her suffering. In contrast, patient B, as stated below, demonstrating a more significant improvement in perceived effectiveness in pain management, tended not to acknowledge these results, focusing more on the fatigue experienced to obtain them. Thus, these results suggest that the attribution of the causes of pain to mental or organic factors can primarily influence the predisposition to listen to one’s internal states and recognize them. The more the attribution of the causes of pain is physical, the more the patient increases the search for external, rather than internal, resolution strategies, with a negative impact on the engagement in the treatment (e.g., in terms of carrying out homework foreseen in the protocol).

Although starting from different assumptions, both patients significantly improved their perceived self-efficacy in pain management and reduced the severity of the affective pain descriptors chosen during the sessions. In patient A, this was related to less need to use all categories to describe pain, selecting only those closely related to the present pain, suggesting a greater awareness of her bodily sensations. In patient B, this was related to achieving essential objectives in carrying out one’s daily activities, reducing the tendency to avoid.

Another relevant mechanism to understand the change process regards the patient’s subjective evaluation of pain and the role of affective and cognitive variables in pain perception (31, 65). Patients who perceive a lack of confidence in their pain management abilities also show negative expectations, a lower sense of agency, and poor investment in different coping strategies, which easily predisposes to a negative evaluation of one’s level of functioning and emotional states characterized by anger, sadness, and fear. On the contrary, a more remarkable ability to regulate one’s emotional states related to chronic pain promotes adaptation to the disease and using flexible and functional strategies for one’s condition (25, 27). These observations are consistent with the results reported in the DERS Scale. Patient A showed an improvement in the subscales related to the tendency to experience negative secondary emotions or non-acceptance reactions in response to one’s distress, the difficulties in maintaining control of one’s behavior, the perception of having limited access to emotion regulation strategies, and lack of clarity to the emotions experienced. Still, as proposed in the protocol, experiential techniques made it possible to observe bodily signals with different attention (e.g., distinguishing, for example, whether what one feels is a painful stimulus or an expression of other experiences). In patient A, during the first sessions, the simple recognition of any internal change was confused with the preamble of something already experienced, such as pain, from which it was necessary to defend oneself and move away as quickly as possible (preventive avoidance strategies). In the last sessions, however, the patient considered how a physical signal did not necessarily determine the onset of unmanageable pain, showing a welcoming and non-judgmental perception of her internal states. This allowed the patient to ‘stay’ with the pain at the proper emotional distance and evaluate which coping strategies might be most functional for her at that moment (shifting of attention—distraction, persistence in carrying out some activities—overdoing, even with a different rhythm—pacing, rest, and use of the drug). Patient B achieved essential goals in managing daily activities, for example, by proceeding with her activities with more functional rhythms and rest phases (pacing strategies). This was also possibly associated with a reduced tendency toward impulsivity in the DERS Scale. Patient B maintained the ability to recognize her internal states while maintaining difficulty in accepting her emotional reactions to exclude any form of vulnerability, including the experience of pain.

Finally, it is interesting to note how the intensity of pain in the last 24 h, measured in the two patients using the BPI Scale, did not show any clinically relevant variation in pre- and post-treatment due to high variability in the ongoing pain in FM patients. Therefore, it would be helpful to consider this variation on a larger timescale. Furthermore, this variability described by patients was perceived as an element capable of worsening their experience of uncertainty and anxiety about the future, a typical response to chronic medical conditions (66, 67). During the INTEGRO clinical protocol, great attention was paid to this aspect, helping patients consider how the prospect of significant variability between 1 day and another allowed them to accommodate even fewer “bad/ugly” moments to carry out different activities. Both patients achieved this objective due to using the MPQ Scale during the clinical sessions. A lower severity and intensity of sensory perceptions and reduced emotional involvement related to the pain experience allowed the two patients to coexist with the pain, reducing feelings of helplessness and the degree of interference with daily activities. This aligns with the reduction observed in the FIQ Scale and the increased sense of self-efficacy in pain management reported on the PSEQ Scale. Techniques focused on pain perception and, more generally, on interoceptive and exteroceptive stimuli enabled patients to learn to ‘be’ with their bodily sensations, perceiving them as less burdensome and catastrophic.

The clinical application of the protocol also allows some reflections regarding its limitations and future developments.

INTEGRO protocol seems promising, although the description of the two reported cases evidences how the potential effectiveness of the intervention depends on some specific characteristics of the patients, such as the subjectivity of pain evaluation. Thus, although considering all the topics covered in the clinical protocol, clinical psychologists must adapt the intervention to the patient’s peculiarities. A good balance needs to be taken between the replicability of the intervention protocol and the need to modulate the number of psychotherapeutic sessions based on the history of the patient with FM, especially as many patients have a history of traumatic experiences (68), which often make body-based intervention more difficult.

INTEGRO intervention is provided only in person, which could be a restriction for patients who cannot move independently, for example, due to worsening pain symptoms. It might be helpful to provide the possibility of consultations through telematic platforms, which have proven useful and effective in breaking down barriers to accessibility and promoting positive outcomes, even in the case of chronic pain and fibromyalgia (39).

Our study aimed to show preliminary results on applying the INTEGRO intervention in the context of fibromyalgia. The high variability observed in the pain and psychological features of patients with fibromyalgia makes it challenging to generalize the findings reported in these cases to the larger clinical population. However, as regards future research, the INTEGRO intervention will be tested in a larger sample of patients with FM to explore its effectiveness and feasibility, and results will allow higher generalizability.

Moreover, applying the INTEGRO intervention on a larger clinical sample would allow us to adapt the intervention based on emerging needs and possible clinical subgroups, which could be categorized by type of pain, coping strategies employed, personality and clinical characteristics, the presence of trauma involving bodily dynamics, the stage of disease acceptance, and the patient’s theories regarding the causes of the illness.

Another possible future evolution of INTEGRO protocol could be the promotion of maintenance groups focused not only on sharing experiences with other patients but also on maintaining the skills learned during the individual path, the integration of sessions dedicated to the creation of an information and educational space led by different specialists in the sector, open to caregivers and patients.

## Data Availability

The raw data supporting the conclusions of this article will be made available by the authors, without undue reservation.
